# Acceptable medication non-adherence: A crowdsourcing study among French physicians for commonly prescribed medications

**DOI:** 10.1371/journal.pone.0209023

**Published:** 2018-12-13

**Authors:** Stéphanie Sidorkiewicz, Viet-Thi Tran, Philippe Ravaud

**Affiliations:** 1 Department of General Medicine, Paris Descartes University, Paris, France; 2 METHODS Team, Centre of Research in Epidemiology and Statistics Sorbonne Paris Cité (CRESS), INSERM, UMR 1153, Paris, France; 3 Cochrane France, Paris, France; 4 Department of Epidemiology, Mailman School of Public Health, Columbia University, New York City, New York, United States of America; Universita degli Studi di Ferrara, ITALY

## Abstract

**Background:**

Achieving good medication adherence is a major challenge for patients with chronic conditions. Our study aimed to assess the Threshold for Unacceptable Risk of Non-adherence (TURN), defined as the threshold at which physicians consider the health risks incurred by patients due to medication non-adherence unacceptable, for the most commonly prescribed drugs in France.

**Methods:**

We conducted an online study using a crowdsourcing approach among French general practitioners and specialists from September 2016 to August 2017. Physicians assessed the TURN for various levels of missed doses by evaluating a series of randomly presented clinical vignettes, each presenting a given medication with a given therapeutic indication. For each “drug-indication group” (i.e., all drugs from the same pharmacological class with a similar therapeutic indication): 1) we described the distribution of physicians’ assessments, 2) we provided a summary estimate of the TURN, defined as the frequency of missed doses above which 75% of the physicians’ assessments were located; 3) we computed the number of pill boxes reimbursed in France in 2016 to put our results into context.

**Results:**

We collected a total of 5365 assessments from 544 physicians, each of whom evaluated a random sample among 528 distinct clinical vignettes. Estimates of the TURN varied widely across drug-indication groups, ranging from risk considered unacceptable with 1 daily dose missed per month (e.g., insulin for diabetes) to risk always considered acceptable (e.g., anti-dementia drugs). Drugs with an estimated TURN of over one missing daily dose per week represented 44.9% of the prescription volume of the medications assessed in our study.

**Conclusions:**

According to physicians, the impact of non-adherence may vary greatly. Patient-physician discussions on the variable consequences of non-adherence could lead to a paradigm shift by seeking to reach “optimal adherence” depending on drugs rather than unrealistic “perfect adherence” to all drugs.

## Introduction

Medication adherence is defined as the extent to which a person’s behavior coincides with medical advice and is often reported as a percentage of the prescribed doses of the medication actually taken by the patient over a specified period [1]. The association between medication non-adherence and clinical outcomes is well described in the literature [1–4]. Adverse events caused by imperfect medication adherence may range from minor symptoms to suboptimal clinical benefit, poor control of the illness [5,6], emergence of treatment resistance [7,8] or life-threatening events [9]. The seriousness of these adverse events depends on the drug and the disease being treated and the magnitude of poor adherence. For example, in the transplantation field, even a minor deviation of optimal adherence to immunosuppressive treatment can lead to severe consequences [10]. For patients with chronic conditions, a large number of studies have found that only 50% to 60% of patients take their medications as prescribed [1,9,11] despite numerous interventions available to improve medication-taking [12]. This finding suggests that for patients taking multiple medications, perfect drug adherence (all doses taken at the correct time, during the whole life) may be unrealistic. Therefore, in clinical practice, physicians seeing patients with various medication-taking behaviors must assess the degree to which non-adherence is acceptable to achieve desired therapeutic effects [13,14], without unnecessarily increasing the burden of treatment on patients [15].

Assessing a threshold of acceptable non-adherence for a large range of medications may have direct implications on clinical practice. Indeed, it may help physicians prioritize their interventions and assess at what point and for which medication they should strongly encourage the patient to change their medication-taking behavior. However, accurately defining a theoretical threshold may be difficult because it must account for the likelihood, clinical consequences and time course of treatment failure [13]. It may also depend on the drug of interest and the medicine-taking behaviors of patients: periodically missing single doses, drug holidays (sequentially multiple missed doses), schedule errors (variability in time of drug intake) or extra doses [11,16,17]. To the best of our knowledge, the effect of medication non-adherence behaviors is only known in very specific therapeutic fields [18,19]. In the literature, the threshold usually used to define “good adherence” is a patient taking more than 80% of all prescribed doses [1,20], but this arbitrary cut-off is commonly applied with no clinical rationale [21] and likely corresponds more to a theoretical threshold for describing populations than to a practical rule used by physicians in clinical practice.

An approach to explore this issue could be to consider the concept of drug “forgiveness” [22,23], that is, the property of a drug, given as a repeated treatment, to forgive the omission of one dose, or several doses in a row, without a loss of efficacy [16,24]. However, forgiveness estimated by pharmacokinetic models [24] only allows to evaluate the short-term risk of adverse events due to poor implementation of a given drug and does not take into account the severity of the adverse event, or the long-term consequences (e.g., poor control of the illness or development of treatment resistance).

In this study, we focused on physicians’ perspectives, who are at the front-line in caring for patients with chronic treatments. They frequently face non-adherence situations, in which they have to use their clinical experience to integrate the different factors involved (the risk of an adverse event, the seriousness of the potential adverse event, the time course of treatment failure [13], the disease being treated) to make clinical decisions. We therefore decided to describe the threshold in terms of frequency of missed doses at which physicians consider the health risks incurred by patients due to medication non-adherence unacceptable for a large range of medications. Although medication adherence should not be reduced to an adherence rate, we considered that this approach would be a first feasible and useful step to explore this complex issue. We hypothesized that physicians’ estimates would not be in line with the 80% threshold usually used in the literature but that on the contrary, they would vary widely among the medications and the diseases.

## Methods

Our study used a crowdsourcing approach by inviting physicians to assess, via the Internet, the Threshold for Unacceptable Risk of Non-adherence (TURN) for the most commonly prescribed medications in France. Physicians determined this threshold for various levels of missed doses by evaluating a series of clinical vignettes, each presenting a given medication in a given chronic indication.

### Definition of the TURN

In this study, we proposed to focus on the new concept of TURN that we defined as the threshold at which physicians consider the health risk incurred by patients due to medication non-adherence unacceptable. For example, for a given medication assessed by a given physician, an estimated threshold of 2 days per month would mean that the physician considered the risk incurred due to non-adherence acceptable as long as patients missed 1 daily dose or less per month. However, 2 daily doses missed per month or more would be unacceptable. Thus, the lower the TURN for a given medication, the more unacceptable physicians consider the health risk due to non-adherence.

### Development of clinical vignettes to assess the TURN

We asked physicians to estimate the TURN for the most commonly prescribed medications in France by using randomly presented clinical vignettes. Each vignette presented one medication (selected according to the number of pill boxes reimbursed by the French national health insurance system during the first 6 months of 2015) in one of its recommended therapeutic chronic indications. The complete procedure of medication selection is described in S1 Fig. For each vignette, physicians estimated the TURN by answering a question inspired from studies describing the consequences of medication non-adherence [5,9,25–28] and methods of risk communication and assessment [29–31]: *“The patient tells you that he/she skips a daily dose of this medication periodically*. *In your opinion*, *at what frequency of missing doses is the risk to his/her health unacceptable*?” Seven answers were proposed, ranging from 1 to 3 days per month (i.e., risk considered unacceptable by physicians with 1 to 3 daily doses missed per month), 1 to 3 days per week, and always acceptable regardless of the frequency. Two other possible responses were “*I do not know*” and “*Other response*”. Respondents were also allowed to write a free comment for each medication assessed. Most of the medications we selected had once-daily dosing regimens, but we adapted the wording of the question to fit the medications with other daily dosing regimens (e.g., 2 or 3 times daily).

A preliminary version of the clinical vignettes was pilot-tested with 4 physicians by using a double interview method. The final question and answers are detailed in S1 Table.

### Data collection

#### Participants

Participants were physicians (general physicians or specialists). We excluded specialists not seeing patients, pediatricians, medical students, residents, and pharmacists. Participants were approached through university or hospital networks (in particular the Société de Formation Thérapeutique du Généraliste–Recherche), congresses (French National Congress of General Medicine and French National Congress of General Medicine Teachers), and social media. Physicians who participated were encouraged to invite their colleagues by a snowball sampling method [32].

Physicians were informed that the data analysis would respect confidentiality. This study was not reviewed by an institutional review board (IRB) because, as a non-interventional study with physicians about their expertise, it did not fall within the scope of French ethics committees. The study has been declared to the French Data Protection Authority (Commission Nationale Informatique et Libertés, CNIL, identification no. 1980063), as required in France. No formal consent was required for this type of study according to French law. Physicians were invited to participate after receiving information about the study on the home page of the study website, and were able to download the study protocol via a dedicated link. Participation by logging into the platform was voluntary and anonymous.

#### Crowdsourcing procedure

Eligible physicians were invited to log into an Internet platform (http://clinicalepidemio.fr/mapp/), where they provided basic demographic information (age and sex) and professional information (medical specialty, ambulatory or hospital practice setting). Then, they assessed as many clinical vignettes as they wanted (they were allowed to skip clinical vignettes for which they did not feel comfortable to assess the TURN). We used algorithms to 1) prevent physicians from assessing only medications from the same pharmacological drug class, and 2) increase the chance that physicians would be evaluating medications from their own specialty first.

### Statistical analysis

Data are presented with numbers (%) for categorical variables and means (SD) for continuous variables. We used complete-case analysis to deal with missing data. All statistical analyses involved the use of R v3.2.2 (http://www.r-project.org).

#### Drug-indication groups

To present our results at an aggregate level, we grouped all drugs from the same pharmacological class in a similar therapeutic indication under a “drug-indication group” according to the 3^rd^ level of the WHO Anatomical Therapeutic Chemical (ATC) classification [33]. Two authors (SS and VT-T) grouped therapeutic indications with the help of specialists in case of uncertainty.

#### Estimation of the TURN for each drug-indication group

**Fig 1:**
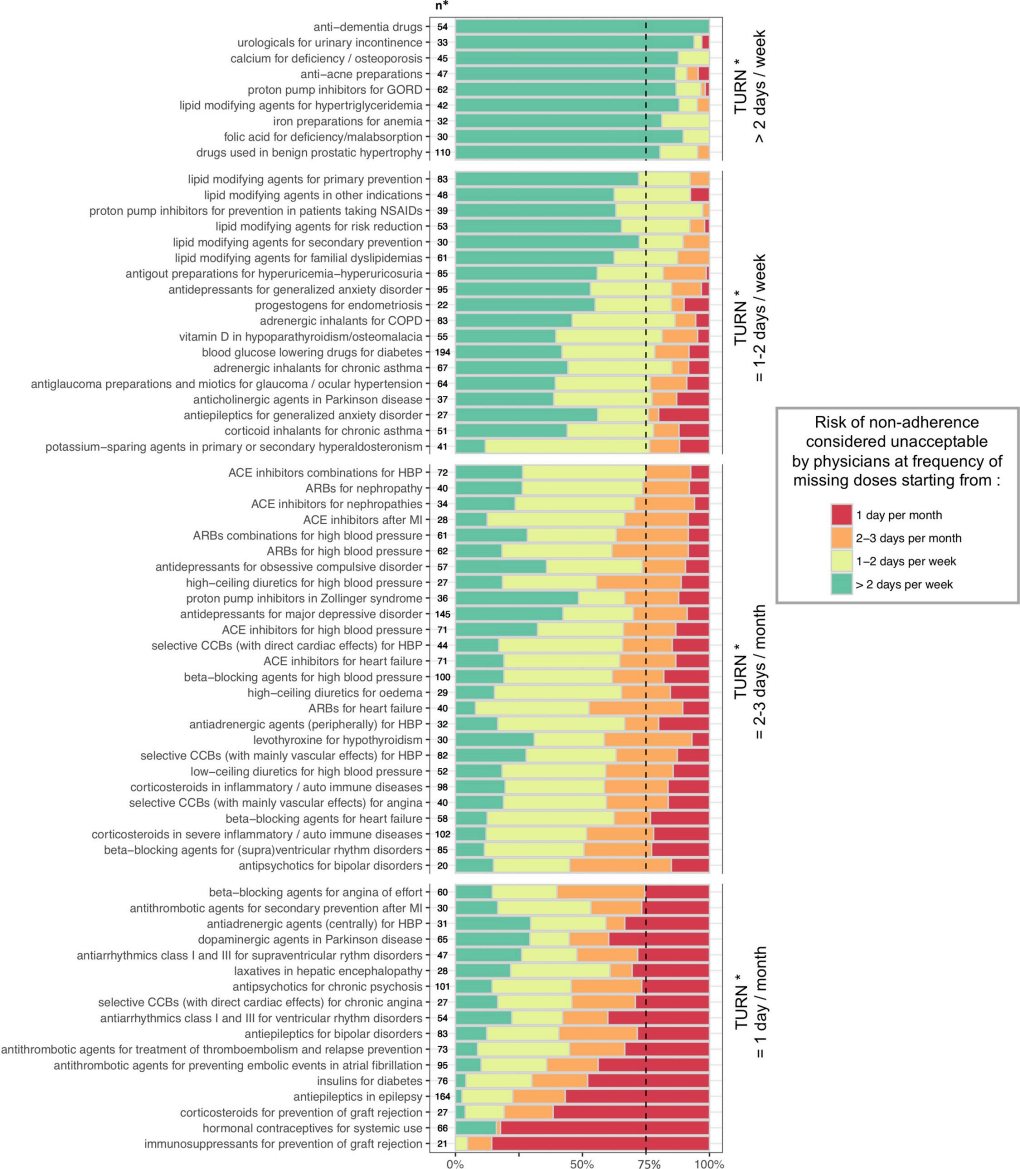
Distribution of physicians’ estimates: Threshold for Unacceptable Risk of Non-Adherence for 70 drug-indication groups. Each horizontal bar represents the distribution (in percentage) of physicians’ estimates for a given drug-indication group. TURN* corresponds to the frequency of missing doses above which 75% of physicians’ estimates were located (vertical dashed line). n* corresponds to the number of physicians’ assessments for each drug-indication group. For sake of clarity, we plotted 70 drug-indication groups corresponding to 90% of total prescription volume of the medications assessed in the study. All the 112 drug-indication groups are plotted in S2 Fig. Abbreviations: ACE: angiotensin-converting enzyme, ARBs: angiotensin receptor blockers, CCBs: calcium channel blockers; COPD: Chronic Obstructive Pulmonary Disease; GORD: gastro-oesophageal reflux diseases; HBP: high blood pressure; NSAIDs: non-steroidal anti-inflammatory drugs, MI: myocardial infarction.

**Fig 2:**
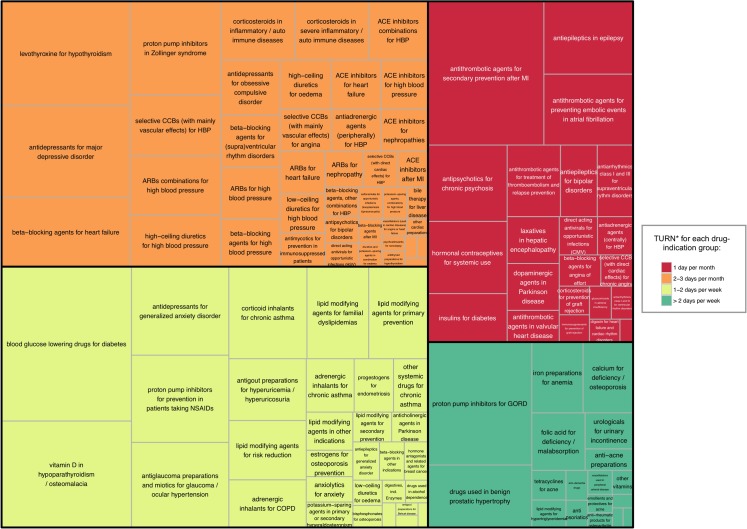
Threshold for Unacceptable Risk of Non-Adherence (TURN) and prescription volume for 112 drug-indication groups. TURN* corresponds to the frequency of missing doses above which 75% of physicians’ estimates were located for each drug-indication group. Results with alternative cut-offs are detailed in S2 Table. Each drug-indication group is plotted as a rectangle: 1) rectangle color corresponds to the TURN*; 2) rectangle area size is proportional to the number of pill boxes reimbursed in 2016 in France (according to the French national health insurance database). We summed the number of pill boxes for the medications belonging to each group. In case of several therapeutic indications for a same medication, we divided the number of pill boxes by the number of indications. Abbreviations: ACE: angiotensin-converting enzyme, ARBs: angiotensin receptor blockers, CCBs: calcium channel blockers; COPD: Chronic Obstructive Pulmonary Disease; GORD: gastro-oesophageal reflux diseases; HBP: high blood pressure; NSAIDs: non-steroidal anti-inflammatory drugs, MI: myocardial infarction.

For each drug-indication group: 1) we plotted the distribution of all physicians’ estimates (Fig 1); 2) we provided a summary estimate of the TURN, that we defined as the frequency of missed doses above which 75% of physicians considered that the health risk incurred by the patients would become unacceptable. For example, if 75% or more physicians considered that missing 2 daily doses or more per month for a given drug-indication group was unacceptable risk, the resulting summary TURN for this drug-indication group was “2 days per month”. No previous data were available about which summary statistic would be clinically relevant to describe the TURN for each drug-indication group. We therefore decided to present our results using a 75% cut-off, based on previous studies assessing Patient Acceptable Symptom States [34] and by adopting a conservative approach for health risk assessment. We performed sensitivity analyses with other cutoffs (more permissive cutoffs: 50%, 60%; and more conservative cutoffs: 80% and 90%).

#### Estimation of the proportion of prescription volume for each drug-indication group

For each drug-indication group, we computed the number of pill boxes reimbursed in 2016 according to the French national health insurance medication database by summing the number of pill boxes for all medications belonging to the given group. We presented our results in a figure that allowed for comparing prescription volumes among the different drug-indication groups and their respective summary TURN: each drug-indication group was presented as a rectangle with color and area size by TURN and prescription volume, respectively (Fig 2).

## Results

### Participants

A total of 544 physicians participated in the study from September 2016 to August 2017. Their mean age was 39.8 years (SD 12.5) and 198 (36.4%) were male. The participants were mainly general physicians (433, 79.6%); 373 (68.6%) worked in ambulatory care and 115 (21.1%) in hospitals. Other characteristics are detailed in Table 1.

**Table 1:** Characteristics of physicians.

Characteristic	Value (N = 544)
Age–mean (SD), years	39.8 (12.5)
Male sex–No. (%)	198 (36.4)
Specialty–No. (%)	
General practitioner	433 (79.6)
Other specialists ^a^	111 (20.4)
Practice setting–No. (%)	
Ambulatory care	373 (68.6)
Hospital	115 (21.1)
Both	37 (6.8)
Other ^b^	19 (3.5)

^a^ other specialists included emergency physicians, rheumatologists, internal medicine specialists, geriatrists, cardiologists, pneumologists, gastroenterologists, psychiatrics, gynecologists, nephrologists, dermatologists, anesthetists, diabetologists.

^b^ not specified.

### Drug-indication groups

A total of 5558 vignettes were submitted to physicians for evaluation, corresponding to 528 distinct clinical vignettes (that is, 528 medications for a given therapeutic indication selected during the process of drug selection detailed in S1 Fig). We collected a total of 5365 assessments (response rate 96.5%) from the 544 physicians, each of whom evaluated a random sample among the 528 vignettes. We grouped all the medications in 124 drug-indication groups. We excluded from the analysis 12 drug-indication groups for which we collected fewer than 10 answers. As a result, we analyzed a total of 112 drug-indication groups. For each of these 112 groups, the mean number of physicians’ assessments was 47.2 (SD 31.5). We also collected 825 free comments from physicians.

### Estimation of the TURN by physicians

We found that the TURN, as assessed by physicians, varied widely across the different drug-indication groups (see Figs 1 and S2). Summary estimates of the TURN ranged from “1 daily dose missed per month” unacceptable for 22 (19.6%) drug-indication groups, to “always acceptable” for 10 (8.9%) groups. The results for other cut-offs are detailed in S2 Table. The three drug-indication groups with the highest TURNs were progestogens and estrogens in combination for menopause, anti-rheumatic products for osteoarthrosis, and anti-dementia drugs (S2 Fig). The three drug-indication groups with the lowest TURNs were immunosuppressants for preventing graft rejection, hormonal contraceptives for systemic use and corticosteroids for preventing graft rejection (S2 Fig).

### Estimating the proportion of prescription volume for each drug-indication group

Fig 2 plots all drug-indication groups with their corresponding summary TURN (rectangle color) and prescription volume (rectangle area size). Overall, 21.0% (in proportion of reimbursed pill boxes) of the medications assessed corresponded to drugs with the lowest TURN (i.e., risk of non-adherence considered unacceptable with 1 daily dose missed per month). Similarly, 44.9% of the medications assessed corresponded to drugs with moderate or high TURN (i.e., risk of non-adherence considered unacceptable with more than 1 missed daily dose per week).

## Discussion

### Principal findings

In this study, we developed the new concept of “Threshold for Unacceptable Risk of Non-adherence” and crowdsourced it from 544 physicians who provided a total 5365 assessments for different drugs in different indications. We found great heterogeneity among physicians in estimating the TURN among the drug classes, with medications for which the estimated TURN was high, such as anti-dementia drugs or dermatological drugs, as compared with medications for which the estimated TURN was low, such as drugs for graft rejection or antiretroviral therapy for HIV infection. The drugs with higher TURNs were mainly those that physicians considered had symptomatic therapeutic indication or poor perceived efficacy. For some of these drugs, physicians stressed the importance of the patient’s decision: “*the patient’s discomfort will guide medication-taking*” (anti-acne preparation); “*the patient is empowered to make his own decision about how to take this medication according to his symptoms*” (drug used for benign prostatic hypertrophy). Some physicians even suggested stopping certain drugs with poor perceived benefit-risk ratio: *“I never initiate this treatment*, *which seems little efficient and with a bad safety profile*. *I advise the patient to stop it”* (anti-dementia drug). In contrast, the drugs with lower TURNs were mainly those with harmful consequences of non-adherence or drugs for which physicians showed accurate knowledge of pharmacokinetics: “*a 12-hour delay exposes the patient to an unwanted pregnancy*” (hormonal contraceptive) and “*the half-life of this drug is short and puts the patient at risk of seizure*” (antiepileptic).

### Strengths and limitations of the study

To the best of our knowledge, this is the first study conducted among physicians about the TURN at a nation-wide level. Crowdsourcing methods allowed us to involve physicians in estimating the TURN for a large amount of different medications among most commonly prescribed medications in France. However, our study has some limitations. First, the physicians were not representative of all French physicians. Second, we presented our results at an aggregate level by pooling medications in drug-indication groups, however, within some groups, the TURN may differ depending on the molecule (e.g., the pharmacokinetic profiles of different vitamin K antagonists are not equivalent). Third, our clinical vignettes were theoretical and did not take into account the patient clinical context, the severity of patients’ conditions or concomitant prescribed drugs, which may explain the variability we found among physicians’ answers for some drug-indication groups (e.g., cardiovascular or antihypertensive drugs). However, for some drug-indication groups, physicians agreed on a TURN with a good consistency across their answers (e.g., drugs against graft rejection). Fourth, our approach was descriptive and does not allow to use the data we collected for future TURN calculation or definition. But the purpose of our study was to explore the physicians’ perspectives about the new concept of the TURN rather than to provide guidelines. Potential gaps with other perspectives (especially patients’) could be interesting to explore in future studies.

### Strengths and limitations in relation to other studies

In the literature, adherence is often described as a ratio of doses taken to the total number of prescribed doses. However, the usual 80% threshold used to define “good adherence” [1,20] may differ for specific treatments such as antiretroviral therapies [1,35]. In line with studies describing the possible heterogeneity across drugs [16,21,36], we considered that medication adherence could not simply be summarized as a unique and identical rate of drug intake for all drugs. However, we did not explore all medication-taking behaviors described in the literature (e.g., drug holidays or extra doses) nor all possible complex adherence patterns [19], which could be further explored in future studies.

Our findings suggest that for 11.4% (in proportion of reimbursed pill boxes) of the medications assessed, the estimated TURN was more than 2 daily missed doses per week. Some physicians even suggested considering non-adherence as a trigger for de-prescribing some medications with poor benefit-risk ratio. These findings are consistent with a recent survey conducted in the United States in which 2106 physicians reported that 20.6% of overall medical care was unnecessary, including 22.0% of prescription medications [37].

## Conclusions

Physicians who are dealing with poor medication adherence in their patients need to decide whether they should intervene to change patients’ behaviors. Considering the TURN may lead to a conceptual shift by changing the definition of “good adherence” and by modifying the care goals for patients taking multiple medications. The care goals for patients taking long-term treatments should no longer be necessarily to achieve “perfect adherence”, without regard to the drug of interest, but rather to seek “optimal adherence.” However, the TURN is only one of the components of decision-making: other aspects may affect the threshold for physicians’ intervention such as the drug’s expected benefit, therapeutic outcomes, patients’ treatment experiences and preferences.

Our study provides a first answer to the question “how much drug non-adherence is acceptable?” However, future research on “how much adherence is enough?” is still needed to help physicians accurately inform their patients, discuss about differential consequences of drug non-adherence, better prioritize interventions, and achieve the desired therapeutic outcomes without putting the patient at risk and without unnecessarily increasing the burden of treatment on patients.

## Supporting information

S1 Fig Development of a list of medications with the corresponding therapeutic indications. (PDF)

S2 Fig Distribution of physicians’ estimates: Threshold for Unacceptable Risk of Non-Adherence (TURN) for 112 drug-indication groups. (PDF)

S1 Table Final version of the questionnaire. (PDF)

S2 Table Sensitivity analyses with different cutoffs to define the summary estimate of the TURN (n = 112 drug-indication groups). (PDF)

S1 File Supporting data 1. (CSV)

S2 File Supporting data 2. (CSV)

S3 File Copy of a clinical vignette used in the study. (PDF)

## References

[1] Osterberg L, Blaschke T. Adherence to medication. N Engl J Med. 2005;353: 487–97. 10.1056/NEJMra050100 16079372 10.1056/NEJMra050100

[2] Dunbar-Jacob J, Mortimer-Stephens MK. Treatment adherence in chronic disease. J Clin Epidemiol. 2001;54 Suppl 1: S57–60. 11750211 10.1016/s0895-4356(01)00457-7

[3] Simpson SH, Eurich DT, Majumdar SR, Padwal RS, Tsuyuki RT, Varney J, et al A meta-analysis of the association between adherence to drug therapy and mortality. BMJ. 2006;333: 15. doi:bmj.38875.675486.55 [pii] 10.1136/bmj.38875.675486.55 16790458 10.1136/bmj.38875.675486.55 PMC1488752

[4] Rasmussen JN, Chong A, Alter DA. Relationship between adherence to evidence-based pharmacotherapy and long-term mortality after acute myocardial infarction. JAMA. 2007;297: 177–86. 10.1001/jama.297.2.177 17213401 10.1001/jama.297.2.177

[5] Ho PM, Rumsfeld JS, Masoudi FA, McClure DL, Plomondon ME, Steiner JF, et al Effect of medication nonadherence on hospitalization and mortality among patients with diabetes mellitus. Arch Intern Med. 2006;166: 1836–1841. 10.1001/archinte.166.17.1836 17000939 10.1001/archinte.166.17.1836

[6] Bramley TJ, Gerbino PP, Nightengale BS, Frech-Tamas F. Relationship of blood pressure control to adherence with antihypertensive monotherapy in 13 managed care organizations. J Manag Care Pharm. 2006;12: 239–45. doi: 10.18553/jmcp.2006.12.3.239 16623608 10.18553/jmcp.2006.12.3.239 PMC10437940

[7] Vanhove GF, Schapiro JM, Winters MA, Merigan TC, Blaschke TF. Patient compliance and drug failure in protease inhibitor monotherapy. JAMA. 1996;276: 1955–6. 8971062

[8] Smith RJ. Adherence to antiretroviral HIV drugs: how many doses can you miss before resistance emerges? Proc Biol Sci. 2006;273: 617–24. 10.1098/rspb.2005.3352 16537134 10.1098/rspb.2005.3352 PMC1560063

[9] Yao X, Abraham NS, Alexander GC, Crown W, Montori VM, Sangaralingham LR, et al Effect of Adherence to Oral Anticoagulants on Risk of Stroke and Major Bleeding Among Patients With Atrial Fibrillation. J Am Heart Assoc. 2016;5 10.1161/JAHA.115.003074 26908412 10.1161/JAHA.115.003074 PMC4802483

[10] Nevins TE, Thomas W. Quantitative patterns of azathioprine adherence after renal transplantation. Transplantation. 2009;87: 711–8. 10.1097/TP.0b013e318195c3d5 19295316 10.1097/TP.0b013e318195c3d5 PMC3580890

[11] Vrijens B, Vincze G, Kristanto P, Urquhart J, Burnier M. Adherence to prescribed antihypertensive drug treatments: longitudinal study of electronically compiled dosing histories. Bmj. 2008;336: 1114–7. 10.1136/bmj.39553.670231.25 18480115 10.1136/bmj.39553.670231.25 PMC2386633

[12] Nieuwlaat R, Wilczynski N, Navarro T, Hobson N, Jeffery R, Keepanasseril A, et al Interventions for enhancing medication adherence. Cochrane Database Syst Rev. 2014; CD000011 10.1002/14651858.CD000011.pub4 25412402 10.1002/14651858.CD000011.pub4 PMC7263418

[13] Steiner JF, Earnest MA. The language of medication-taking. Ann Intern Med. 2000;132: 926–30. 10836931 10.7326/0003-4819-132-11-200006060-00026

[14] Gulick RM. Adherence to antiretroviral therapy: how much is enough? Clin Infect Dis. 2006;43: 942–4. 10.1086/507549 16941381 10.1086/507549

[15] Tran VT, Montori VM, Eton DT, Baruch D, Falissard B, Ravaud P. Development and description of measurement properties of an instrument to assess treatment burden among patients with multiple chronic conditions. BMC Med. 2012;10: 68 10.1186/1741-7015-10-68 22762722 10.1186/1741-7015-10-68 PMC3402984

[16] Osterberg LG, Urquhart J, Blaschke TF. Understanding forgiveness: minding and mining the gaps between pharmacokinetics and therapeutics. Clin Pharmacol Ther. 2010;88: 457–9. 10.1038/clpt.2010.171 20856243 10.1038/clpt.2010.171

[17] Sidorkiewicz S, Tran VT, Cousyn C, Perrodeau E, Ravaud P. Development and validation of an instrument to assess treatment adherence for each individual drug taken by a patient. BMJ Open. 2016;6: e010510 10.1136/bmjopen-2015-010510 27165645 10.1136/bmjopen-2015-010510 PMC4874131

[18] Urquhart J. How much compliance is enough? Pharm Res. 1996;13: 10–1. 8668655 10.1023/a:1016004611847

[19] Maclean JR, Pfister M, Zhou Z, Roy A, Tuomari VA, Heifets M. Quantifying the impact of nonadherence patterns on exposure to oral immunosuppressants. Ther Clin Risk Manag. 2011;7: 149–56. 10.2147/TCRM.S16870 21691585 10.2147/TCRM.S16870 PMC3116802

[20] Schroeder K, Fahey T, Ebrahim S, Peters TJ. Adherence to long-term therapies: recent WHO report provides some answers but poses even more questions. J Clin Epidemiol. 2004;57: 2–3. 10.1016/j.jclinepi.2003.07.002 15019004 10.1016/j.jclinepi.2003.07.002

[21] Stauffer ME, Hutson P, Kaufman AS, Morrison A. The Adherence Rate Threshold is Drug Specific. Drugs R D. 2017;17: 645–653. 10.1007/s40268-017-0216-6 29076037 10.1007/s40268-017-0216-6 PMC5694429

[22] Morrison A, Stauffer ME, Kaufman AS. Relationship Between Adherence Rate Threshold and Drug “Forgiveness.” Clin Pharmacokinet. 2017; 10.1007/s40262-017-0552-2 28470545 10.1007/s40262-017-0552-2

[23] Assawasuwannakit P, Braund R, Duffull SB. Quantification of the Forgiveness of Drugs to Imperfect Adherence. CPT Pharmacometrics Syst Pharmacol. 2015;4: e00004 10.1002/psp4.4 26225235 10.1002/psp4.4 PMC4394614

[24] Boissel JP, Nony P. Using pharmacokinetic-pharmacodynamic relationships to predict the effect of poor compliance. Clin Pharmacokinet. 2002;41: 1–6. 10.2165/00003088-200241010-00001 11825093 10.2165/00003088-200241010-00001

[25] Denhaerynck K, Burkhalter F, Schäfer-Keller P, Steiger J, Bock A, De Geest S. Clinical consequences of non adherence to immunosuppressive medication in kidney transplant patients. Transpl Int. 2009;22: 441–6. 10.1111/j.1432-2277.2008.00820.x 19144090 10.1111/j.1432-2277.2008.00820.x

[26] Prihodova L, Nagyova I, Rosenberger J, Majernikova M, Roland R, Groothoff JW, et al Adherence in patients in the first year after kidney transplantation and its impact on graft loss and mortality: a cross-sectional and prospective study. J Adv Nurs. 2014;70: 2871–83. 10.1111/jan.12447 24853863 10.1111/jan.12447

[27] Emsley R. Non-adherence and its consequences: understanding the nature of relapse. World Psychiatry. 2013;12: 234–5. 10.1002/wps.20067 24096786 10.1002/wps.20067 PMC3799251

[28] Ho PM, Bryson CL, Rumsfeld JS. Medication adherence: its importance in cardiovascular outcomes. Circulation. 2009;119: 3028–35. 10.1161/CIRCULATIONAHA.108.768986 19528344 10.1161/CIRCULATIONAHA.108.768986

[29] Opel DJ, Taylor JA, Phillipi CA, Diekema DS. The intersection of evidence and values in clinical guidelines: who decides what constitutes acceptable risk in the care of children? Hosp Pediatr. 2013;3: 87–91. 24340407 10.1542/hpeds.2012-0090

[30] Devereaux PJ, Anderson DR, Gardner MJ, Putnam W, Flowerdew GJ, Brownell BF, et al Differences between perspectives of physicians and patients on anticoagulation in patients with atrial fibrillation: observational study. Bmj. 2001;323: 1218–22. 11719412 10.1136/bmj.323.7323.1218 PMC59994

[31] Fischhoff B, Slovic P, Lichtenstein S, Read S, Combs B. How safe is safe enough? A psychometric study of attitudes towards technological risks and benefits. Policy Sciences. 1978;9: 127–152. 10.1007/bf00143739

[32] Goodman Lea A. Snowball Sampling. Annals of Mathematical Statistics. 1961;32: 148–170.

[33] WHO Collaborating Centre for Drug Statistics Methodology. ATC/DDD Index 2017 [Internet]. 2017. Available: https://www.whocc.no/atc_ddd_index/

[34] Tubach F, Ravaud P, Baron G, Falissard B, Logeart I, Bellamy N, et al Evaluation of clinically relevant states in patient reported outcomes in knee and hip osteoarthritis: the patient acceptable symptom state. Ann Rheum Dis. 2005;64: 34–7. 10.1136/ard.2004.023028 15130902 10.1136/ard.2004.023028 PMC1755171

[35] Paterson DL, Swindells S, Mohr J, Brester M, Vergis EN, Squier C, et al Adherence to protease inhibitor therapy and outcomes in patients with HIV infection. Ann Intern Med. 2000;133: 21–30. 10877736 10.7326/0003-4819-133-1-200007040-00004

[36] Wu JR, Moser DK, De Jong MJ, Rayens MK, Chung ML, Riegel B, et al Defining an evidence-based cutpoint for medication adherence in heart failure. Am Heart J. 2009;157: 285–91. 10.1016/j.ahj.2008.10.001 19185635 10.1016/j.ahj.2008.10.001 PMC2668834

[37] Lyu H, Xu T, Brotman D, Mayer-Blackwell B, Cooper M, Daniel M, et al Overtreatment in the United States. PLoS One. 2017;12: e0181970 10.1371/journal.pone.0181970 28877170 10.1371/journal.pone.0181970 PMC5587107

