# Supplementary material for: Acceptable medication non-adherence: A crowdsourcing study among French physicians for commonly prescribed medications
Source: PLoS One. 2018 Dec 13;13(12):e0209023. doi: 10.1371/journal.pone.0209023 (PMC6292617; doi:10.1371/journal.pone.0209023)
Supplement: S1 Fig — (PDF) [file pone.0209023.s001.pdf]

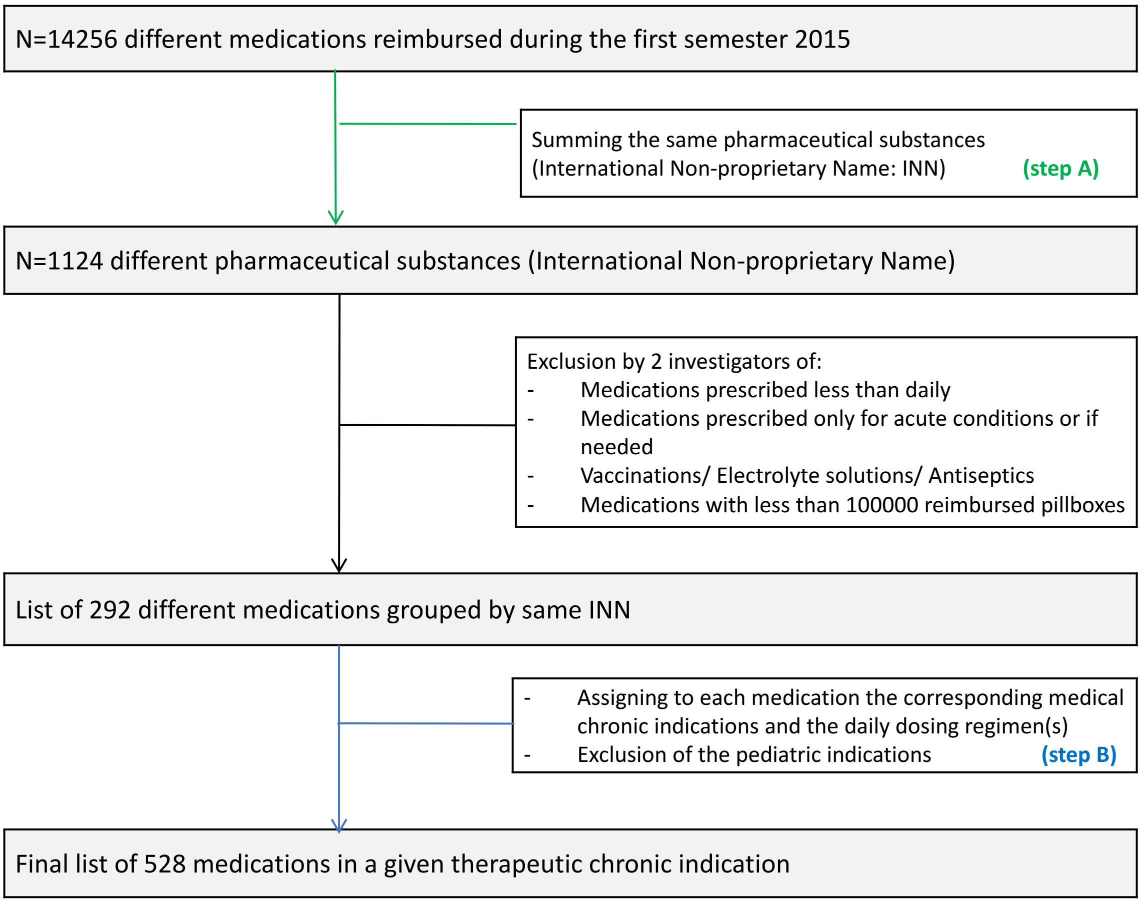

Step A: Example of medication grouping process

Initial List of medications	
**Commercial Name**	Substance (INN convention)
**STAGID**	METFORMIN
**GLUCOPHAGE**	METFORMIN

→

List of medications after regrouping by substance	
**Commercial Names**	Substance (INN convention)
**STAGID, GLUCOPHAGE**	METFORMIN

Step B: Example of assignment of therapeutic indication

List of medications after regrouping by substance	
**Commercial Names**	Substance (INN convention)
**STAGID, GLUCOPHAGE**	METFORMIN
**AVLOCARDYL**	PROPANOLOL

→

List of medications assigned to corresponding therapeutic indications		
**Commercial Names**	Substance (INN convention)	Medical Indication
**STAGID, GLUCOPHAGE**	METFORMIN	Diabetes treatment
**AVLOCARDYL**	PROPANOLOL	Long-term treatment after myocardial infarction
**AVLOCARDYL**	PROPANOLOL	High blood pressure treatment
**AVLOCARDYL**	PROPANOLOL	Migraine long-term treatment
**AVLOCARDYL**	PROPANOLOL	Supraventricular rhythm disorders treatment

S1 Fig. Development of a list of medications with the corresponding therapeutic indications.
