# Supplementary material for: Acceptable medication non-adherence: A crowdsourcing study among French physicians for commonly prescribed medications
Source: PLoS One. 2018 Dec 13;13(12):e0209023. doi: 10.1371/journal.pone.0209023 (PMC6292617; doi:10.1371/journal.pone.0209023)
Supplement: S2 Fig — (PDF) [file pone.0209023.s002.pdf]

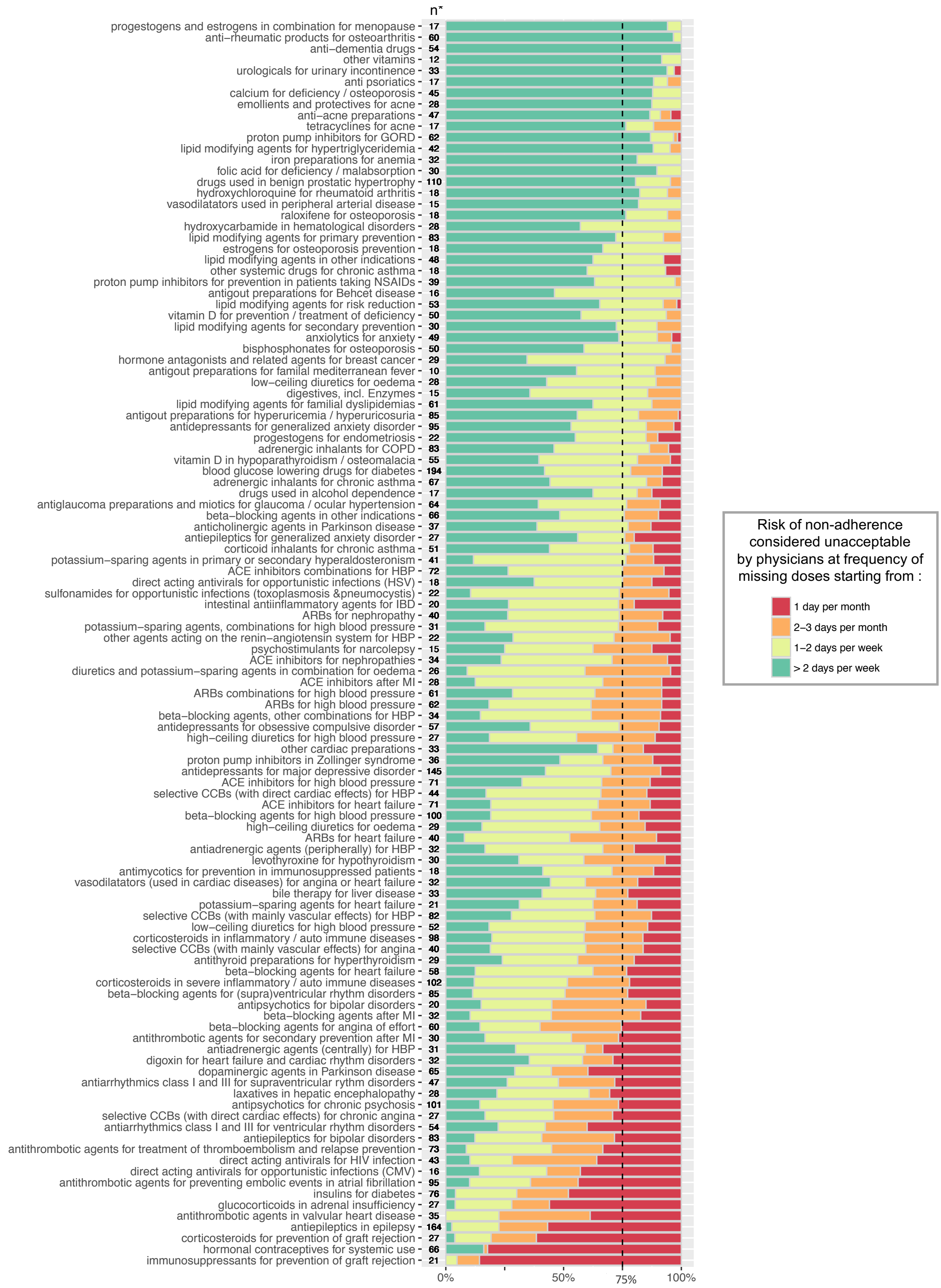


## S2 Fig. Distribution of physicians' estimates : Threshold for Unacceptable Risk of Non-Adherence (TURN) for 112 drug-indication groups.

Each horizontal bar represents the distribution (in percentage) of physicians' estimates of the TURN for a given drug-indication group. The vertical dashed line corresponds to the frequency of missing doses over which 75% of physicians' estimates were located.

* n corresponds to the number of physicians' assessments for each drug-indication group.

Abbreviations: ACE: angiotensin-converting enzyme, ARBs: angiotensin receptor blockers, CCBs: calcium channel blockers; COPD: Chronic Obstructive Pulmonary Disease; GORD: gastro-oesophageal reflux diseases; HBP: high blood pressure; NSAIDs: non-steroidal anti-inflammatory drugs, MI: myocardial infarction
