# Supplementary material for: Acceptable medication non-adherence: A crowdsourcing study among French physicians for commonly prescribed medications
Source: PLoS One. 2018 Dec 13;13(12):e0209023. doi: 10.1371/journal.pone.0209023 (PMC6292617; doi:10.1371/journal.pone.0209023)
Supplement: S1 Table — (PDF) [file pone.0209023.s003.pdf]

S1 Table. Final version of the questionnaire.

The patient tells you that he/she skips a daily dose of this medication periodically. In your opinion, at what frequency of missing doses is the risk to his/her health unacceptable? From missing daily doses occurring:

- One day per month
- Two days per month
- Three days per month
- One day per week
- Two days per week
- Three days per week
- The risk is always acceptable regardless the frequency of missing doses
- Other response:
- I do not know
