# Supplementary material for: Acceptable medication non-adherence: A crowdsourcing study among French physicians for commonly prescribed medications
Source: PLoS One. 2018 Dec 13;13(12):e0209023. doi: 10.1371/journal.pone.0209023 (PMC6292617; doi:10.1371/journal.pone.0209023)
Supplement: S2 Table — (PDF) [file pone.0209023.s004.pdf]

S2 Table. Sensitivity analyses with different cutoffs to define the summary estimate of the TURN (n=112 drug-indication groups).

	TURN: frequency of missing doses (days)						Always acceptable
**Cutoff ***	**1/month -n**	**2/month -n**	**3/month -n**	**1/week -n**	**2/week -n**	**3/week -n**	**-n**
**50% -n**	5 [a]	5	10	35	20	13	24
**60% -n**	10	10	16	31	17	9	19
**75% -n**	22	16	25	20	11	8	10
**80% -n**	28	21	21	22	4	8	8
**90% -n**	56	17	11	17	6	0	5 [b]

n corresponds to the number of drug-indication groups
* the cutoff corresponds to the percentage used to define the summary estimate of the TURN (e.g., for the cutoff highlighted in grey [75%] in the primary analysis, the summary TURN was defined as response category over which 75% of physicians' estimates were located).

[a] Drug-indication groups for which the TURN is "1 day/month" whatever the cut-off: antiepileptics in epilepsy, corticosteroids for prevention against graft rejection, glucocorticoids in adrenal insufficiency, hormonal contraceptives for systemic use, immunosuppressants for prevention against graft rejection

[b] Drug-indication groups for which the TURN is "always acceptable" whatever the cut-off: anti-dementia drugs, anti-rheumatic products for osteoarthritis, other vitamins, urologics for urinary incontinence, progestogens and estrogens in combination for menopause
