# Supplementary material for: Acceptable medication non-adherence: A crowdsourcing study among French physicians for commonly prescribed medications
Source: PLoS One. 2018 Dec 13;13(12):e0209023. doi: 10.1371/journal.pone.0209023 (PMC6292617; doi:10.1371/journal.pone.0209023)
Supplement: S3 File — (PDF) [file pone.0209023.s007.pdf]

S3 File. Copy of a clinical vignette used in the study

Copy of one of the 528 vignettes used in the study (in French)

MAPP-Study | Accueil | L'étude | L'équipe | Contact | Espace personnel

Se déconnecter

3/10

Un patient qui ne prend pas un traitement prescrit s'expose à un risque potentiel, variable selon le médicament.

Merci d'évaluer la situation clinique ci-dessous :

Un de vos patients est traité par :
PREDNISOLONE (SOLUPRED) 1 prise le matin
pour :
Prophylaxie ou traitement du rejet de greffe.

Le traitement a été initié il y a plus de 3 mois. Les pathologies du patient sont équilibrées.

Le patient vous apprend qu'il saute une prise de ce médicament de temps en temps.
Selon vous, quand le risque encouru pour sa santé devient-il inacceptable ?

Pour un saut d'une prise survenant :

- 1 jour par mois
- 2 jours par mois
- 3 jours par mois
- 1 jour par semaine
- 2 jours par semaine
- 3 jours par semaine
- Risque toujours acceptable quelque soit la fréquence des sauts
- Je propose l'arrêt du traitement car la balance bénéfice-risque me semble peu favorable dans cette indication
- Autre :
- Je ne sais pas

English translation

MAPP-Study | Home | About the study | Research team | Contact | Personal

Log out

A patient who does not take a prescribed medication is exposed to a potential risk, which varies depending on the medication. Please evaluate the clinical situation below:
One of your patients is treated by:
PREDNISOLONE (SOLUPRED): one dose in the morning
For:
Preventing graft rejection.
The treatment was initiated more than 3 months ago. The patient's conditions are stable.

The patient tells you that he/she skips a daily dose of this medication periodically. In your opinion, at what frequency of missing doses is the risk to his/her health unacceptable?
From missing daily doses occurring:

- One day per month
- Two days per month
- Three days per month
- One day per week
- Two days per week
- Three days per week
- The risk is always acceptable regardless the frequency of missing doses
- I suggest discontinuing the treatment based on its risk-benefit ratio.
- Other response:
- I do not know
